# Supplementary material for: Immune Alterations in a Patient with SARS-CoV-2-Related Acute Respiratory Distress Syndrome
Source: J Clin Immunol. 2020 Aug 22;40(8):1082–92. doi: 10.1007/s10875-020-00839-x (PMC7443154; doi:10.1007/s10875-020-00839-x)
Supplement: Supplementary file 1 — (PDF 85 kb) [file 10875_2020_839_MOESM1_ESM.pdf]

Immune alterations in a patient with SARS-CoV-2-related acute respiratory distress syndrome

Lila Bouadma[1,2 a], Aurélie Wiedemann[3 a], Juliette Patrier[1], Mathieu Surénaud[3], Paul-Henri Wicky[1], Emile Foucat[3], Jean-Luc Diehl[4], Boris P. Hejblum[5], Fabrice Sinnah[1], Etienne de Montmollin[1], Christine Lacabaratz[3], Rodolphe Thiébaut[5,6], JF Timsit[1,2 b] and Yves Lévy[3,7 b *]

Affiliations

1. APHP- Hôpital Bichat – Médecine Intensive et Réanimation des Maladies Infectieuses, Paris, France

2. UMR 1137 - IAME Team 5 – DeSCID: Decision Sciences in Infectious Diseases, Control and Care, Inserm/Univ Paris Diderot, Sorbonne Paris Cité, Paris, France

3. Vaccine Research Institute, Université Paris-Est Créteil, Faculté de Médecine, INSERM U955, Créteil, France

4. APHP, Hôpital Georges Pompidou, Médecine Intensive Reanimation, Paris France

5. Univ. Bordeaux, Department of Public Health, Inserm Bordeaux Population Health Research Centre, Inria SISTM, UMR 1219; Vaccine Research Institute (VRI), Créteil, France

6. CHU Bordeaux, Bordeaux, France

7. Assistance Publique-Hôpitaux de Paris, Groupe Henri-Mondor Albert-Chenevier, Service Immunologie Clinique, Créteil, France

a: co-first authors

b: co-last authors

***Correspondence:** Pr. Yves Lévy, E-mail: yves.levy@aphp.fr

Supplementary Table 1 : Polynuclear neutrophil counts (PNN) in the blood

**Day of sampling (D)**	**PNN (/mm^3)<br>[N.V: 2000-8000/mm^3]**
D14	17600
D15	10000
D16	7700
D20	8700
D21	15000
D23	19600
D24 (death)	N.D

N.V : normal values, N.D : not determined
